# Predictive factors of non-adherence to secondary preventative medication after stroke or transient ischaemic attack: A systematic review and meta-analyses

**DOI:** 10.1177/2396987316647187

**Published:** 2016-05-05

**Authors:** Sukainah Al AlShaikh, Terry Quinn, William Dunn, Matthew Walters, Jesse Dawson

**Affiliations:** Institute of Cardiovascular and Medical Sciences, College of Medical, Veterinary and Life Sciences, University of Glasgow, Glasgow, UK

**Keywords:** Stroke, medication, adherence, prevalence, predictors, transient ischaemic attack

## Abstract

**Purpose:**

Non-adherence to secondary preventative medications after stroke is relatively common and associated with poorer outcomes. Non-adherence can be due to a number of patient, disease, medication or institutional factors. The aim of this review was to identify factors associated with non-adherence after stroke.

**Method:**

We performed a systematic review and meta-analysis of studies reporting factors associated with medication adherence after stroke. We searched MEDLINE, EMBASE, CINAHL, PsycINFO, CENTRAL and Web of Knowledge. We followed PRISMA guidance. We assessed risk of bias of included studies using a pre-specified tool based on Cochrane guidance and the Newcastle–Ottawa scales. Where data allowed, we evaluated summary prevalence of non-adherence and association of factors commonly reported with medication adherence in included studies using random-effects model meta-analysis.

**Findings:**

From 12,237 titles, we included 29 studies in our review. These included 69,137 patients. The majority of included studies (27/29) were considered to be at high risk of bias mainly due to performance bias. Non-adherence rate to secondary preventative medication reported by included studies was 30.9% (95% CI 26.8%–35.3%). Although many factors were reported as related to adherence in individual studies, on meta-analysis, absent history of atrial fibrillation (OR 1.02, 95% CI 0.72–1.5), disability (OR 1.27, 95% CI 0.93–1.72), polypharmacy (OR 1.29, 95% CI 0.9–1.9) and age (OR 1.04, 95% CI 0.96–1.14) were not associated with adherence.

**Discussion:**

This review identified many factors related to adherence to preventative medications after stroke of which many are modifiable. Commonly reported factors included concerns about treatment, lack of support with medication intake, polypharmacy, increased disability and having more severe stroke.

**Conclusion:**

Understanding factors associated with medication taking could inform strategies to improve adherence. Further research should assess whether interventions to promote adherence also improve outcomes.

## Introduction

It is recognised that adherence to secondary preventative medications after stroke is variable; in some studies more than half of participants stopped taking their prescribed drugs 1–2 years after the stroke incident.^1–3^ Use of the secondary prevention strategies has been reported to result in 80% reduction in the risk of stroke recurrence, vascular events or death^4,5^ and poor adherence is related to adverse outcomes.^6–8^

Many factors interfere with the ability of stroke patients to regularly take their medications. Stroke survivors may have disability or cognitive issues which make them unable to self-administer medication.^9–11^ Personal beliefs and preferences may also impact adherence.^10^ Medication factors also affect adherence. Drugs such as anti-coagulants typically have less adherence than anti-platelets^11^ and cost of medications is also of potential importance.^9^ Health care system failure exists through lack of access to health care and inadequate communication with health care providers.^12^

Several studies have attempted to identify barriers to adherence to medication after stroke. Patients with stroke expressed that concerns about prescribed medication and unawareness of the rationale of treatment as primary reasons for non-adherence.^13^ We performed a systematic review and meta-analysis of studies that assessed predictive factors for adherence to preventative medications in patients with stroke or transient ischaemic attack (TIA).

## Methodology

We performed a systematic review and meta-analysis following Preferred Reporting Items for Systematic Reviews and Meta-analyses (PRISMA) guidelines^14^ for design, conduct and reporting. The review protocol was registered in PROSPERO (registration number: CRD42015027531).

### Search strategy and study selection

We generated search strings based on concepts of ‘Stroke’ and ‘Medication Adherence.’ We focussed on MeSH terms and other controlled vocabulary (available in the supplementary appendix, which can be found online with this review). Two independent reviewers (SA and WD) searched Web of Knowledge, EMBASE, MEDLINE (both using Ovid), CINAHL, PsycINFO (both in EBSCOhost) and CENTRAL (Cochrane Library). Initially, titles were reviewed and possibly eligible articles were listed for abstract review. These were then retrieved for entire text review by SA. We also reviewed reference lists of included studies and related reviews to detect additional reports.

### Eligibility criteria

We only included studies published in English. Studies had to include adults (aged ≥ 18 years) who had suffered stroke or TIA and were prescribed medication for the prevention of recurrent cardiovascular events. Studies had to assess factor(s) that influenced medication adherence. Where disagreement arose regarding study eligibility, a consensus meeting was arranged with an arbitrator (JD). We excluded from this review studies that did not include a measure of medication adherence, studies that assessed non-pharmacological preventative strategies only or did not include stroke or TIA patients.

### Data extraction

We designed a data extraction form that summarised information on study characteristics, inclusion criteria, sample size, secondary preventative medications, method used to measure adherence and predictive factors. We did not contact the study authors for missing information or for clarification.

### Assessment of risk of bias in included studies

We assessed risk of bias in included studies using a pre-specified tool generated using Cochrane Library tool for assessing risk of bias^15^ and the Newcastle–Ottawa scales.^16^ Two independent reviewers (SA and JD) assessed risk of bias and met to finalise the assessment. Disagreement was resolved via discussion until reaching a mutual agreement. We considered studies as of high quality if they met the criteria for all the assessment domains (selection, performance, attrition, reporting and confounders).

### Data synthesis and analysis

We categorised preventative medications as anti-coagulants, anti-platelet, blood pressure or lipid lowering drugs. Some studies also reported adherence to the overall medication regimen without specification of medication classes. We listed predictive factors, significance (odds or hazard ratios and 95% confidence intervals) and the type of analysis used. We used the World Health Organization (WHO) classification of predictive factors of non-adherence, which categorised these into five domains:^17^

– Patient related factors
– Social and economic related factors
– Therapy-related factors
– Health system or health care team related factors and
– Condition (stroke)-related factors

We described included studies and factors reported to be significant using a narrative review. Where a factor was assessed in more than three studies we described a summary value using random-effects models meta-analyses. We also described summary measures of medication non-adherence across non-case control studies. These analyses used Comprehensive Meta-Analysis software (CMA, version 2.0, Biostat Inc).

## Results

**Figure 1.**
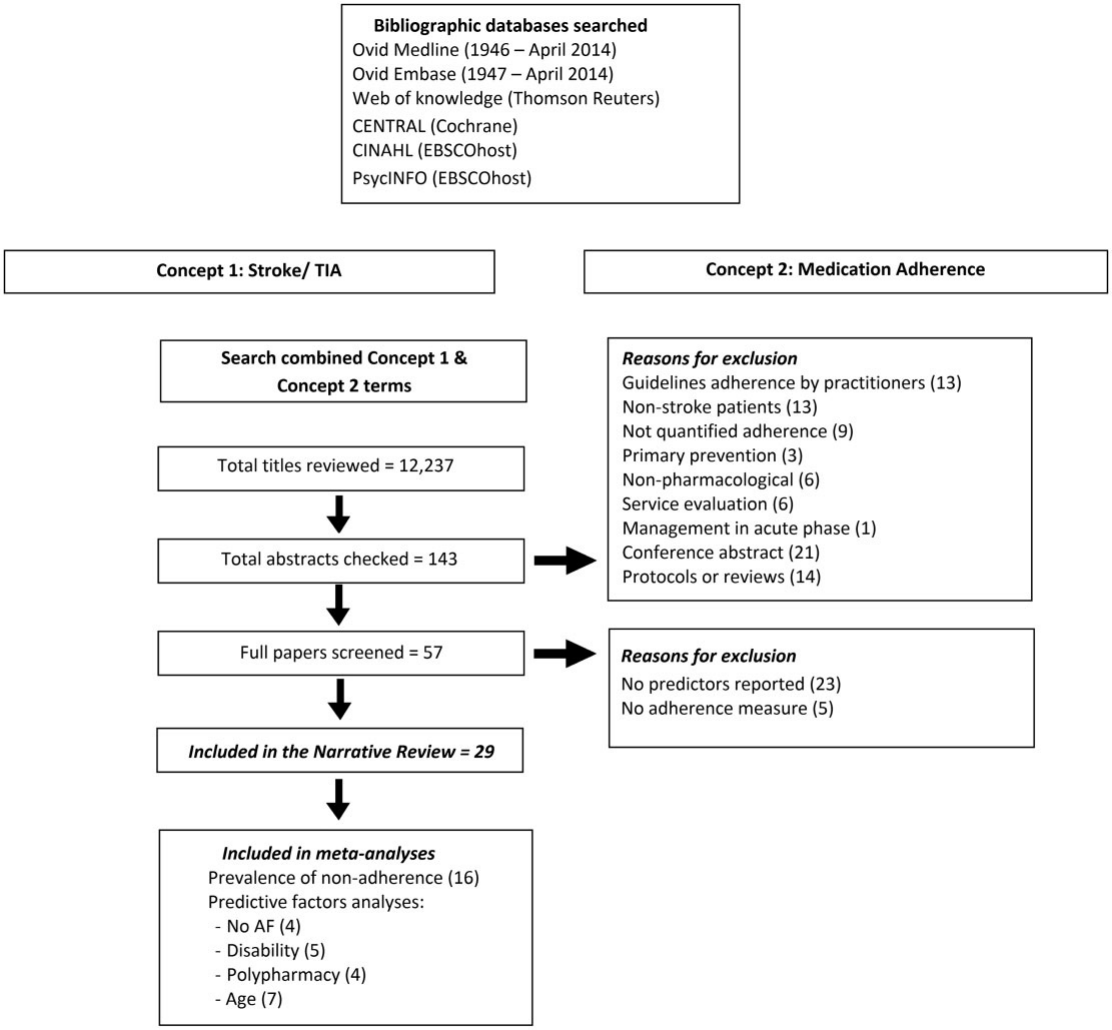
PRISMA flow diagram.

The search was completed in April 2014 and identified a total of 12,237 titles. Title review identified 143 papers for abstract review. Of these 57 were retrieved for full-text review. We identified 29 of these as meeting our eligibility criteria (Figure 1).^1,2,9–12,18–40^

### Risk of bias across included studies

Studies included in this review were all of high risk of bias (except two^34,36^) mainly because details on performance bias, represented by blinding of outcome assessor, were not reported. It was also unclear whether there was a selective reporting of the outcomes in a study.^23^ Twelve studies were non-controlled.^2,9,10,18–20,22,28,32,38–40^ In addition, most studies used a subjective method to monitor adherence which has been reported to overestimate patients’ adherence.^41,42^ More details on other sources of bias in included studies are available in the supplementary appendix.

### Narrative review

#### Description of eligible studies

The 29 included studies were observational studies of which 14 were prospective cohorts,^1,2,9,10,18,20,24,26,32,35,36,38–40^ 4 were retrospective cohorts,^22,28,33,34^ 9 used a cross-sectional design^11,12,21,25,27,29–31,37^ and two performed a case-control analysis.^19,23^ Details of study characteristics can be found in Table 1. The total number of participants in the included studies was 69,137. Reported non-adherence rate ranged between 11.3%^39^ and 45.2%.^30^

**Table 1.** Characteristics of included studies.

Study	Design	Inclusion criteria	Exclusion criteria	Sample size	Medication classes	Adherence assessment measure
Arif et al.^21^	Cross-sectional	First-time stroke	MI Non-ischaemic or non-haemorrhagic TIA	298	AP AH LLD	Telephone interview
Burke et al.^22^	Retrospective cohort	First-time IS	Previous cardiac condition Previous AT	1413	AP	Prescription refill
Bushnell et al.^18^	Observational cohort, 3 months	IS or TIA	–	2598	AP AC AH LLD	Telephone interview
Bushnell et al.^18^	Longitudinal study, 1 year	IS or TIA	–	2457	AP AC AH LLD	Telephone interview
Chambers et al.^23^	Case-control study	First- time IS	Institutional living	26	Not specified	MARS and BMQ
Choi-Kwon et al.^24^	Observational cohort, 1–5 years	Early-onset stroke patients (onset between ages of 15–45 years)	HS TIA Severe medical conditions Previous stroke	256	AH	Patient interview
Coetzee et al.^25^	Cross-sectional at 6 weeks	Completed rehabilitation program	–	26 (compared to 29 amputee patients)	All classes	Patient interview and pill count
De Schryver et al.^26^	Cohort study, 1–2 years	Patients in the Dutch TIA Trial and the Stroke Prevention In Reversible Ischaemia Trial	–	3796 (aspirin) and 651 (AC)	Aspirin AC	Patient interview and pill count
Edmondson et al.^27^	Cross-sectional	Age > 40 years Stroke or TIA	Institutional living Pregnant Aphasia Cognitive impairment	535	AT AH LLD	MMAS and BMQ
Glader et al.^2^	Prospective observational study, 2 year	Patients in the Swedish Stroke Register	–	24,024	AP AC AH LLD	Prescription refill
Huang et al.^28^	Retrospective cohort, 1 year	IS or TIA	In-hospital stroke	11,050	AT AH LLD	Prescription refill
Ji et al.^29^	Cross-sectional, at 3 months	IS or TIA	–	9998	AP AC AH LLD	Telephone interview
Ke et al.^30^	Cross-sectional	Cerebral infarction TIA	–	1240	Aspirin	Telephone interview
Kronish et al.^31^	Cross-sectional	Stroke or TIA in the past 5 years	Institutional living Pregnant Aphasia Cognitive impairment	535	Not specified	MMAS
Kronish et al.^12^	Cross-sectional study	Stroke or TIA Age ≥ 40 years	Aphasia Cognitive impairment Pregnant Institutional living	600	Not specified	MMAS
Levine et al.^19^	Case-control study	Stroke Age ≥ 45 years Noninstitutionalized	–	8673	Not specified	Questionnaire
Lopes et al.^32^	Longitudinal study, 1 year	IS or TIA with AF in Get With The Guidelines (GWTG)–Stroke registry & Adherence eValuation After Ischemic Stroke Longitudinal (AVAIL) registry	Bleeding Palliative-care Death or transfer from hospital	291	AC	Patient interview
Lummis et al.^9^	Cohort study, 1 year	Stroke patients in the Stroke Outcome Study	–	420	AT AH LLD	Self-reported adherence
O’Carroll et al.^10^	Longitudinal study, 1 year	First-time IS Responsible for own medication	Institutional living	180	AH Aspirin LLD	MARS, BMQ and urinary- salicylate level
Østergaard et al.^33^	Retrospective cohort	Suspected stroke	HS	503	AP	Prescription refill
Østergaard et al.^34^	Retrospective cohort, 1.7 years	TIA	Prior TIA or stroke & previous AC	594	AP	Prescription refill
Rodriguez et al.^35^	Longitudinal study, 1 year	IS or TIA GWTG-Stroke program	–	2720	AP AC AH LLD	Telephone interview
Sappok et al.^36^	Prospective observational study, 1 year	IS or TIA	Haemorrhage Migraine Epilepsy	470	AT	Telephone interview
Sjölander et al.^38^	Prospective observational study	Ischemic stroke in the Swedish Stroke Register	–	18,349	AH	Medication refill
Sjölander et al.^37^	Cross-sectional	Stroke	Institutional-living	578	Not specified	MARS
Thrift et al.^20^	Prospective cohort, 10 years	Stroke	Subarachnoid haemorrhage	1241	AT AH LLD	Self-reported adherence
Wang et al.^11^	Cross-sectional, at 1 year	TIA or a cerebral infarction	Haemorrhage Migraine Epilepsy	722	AT	Telephone interview
Weimar et al.^39^	Observational cohort, 1–2 years	Cerebrovascular disease with AF	Intracerebral haemorrhage	293	AC	Patient interview
Xu et al.^40^	Prospective cohort, 1-year	Stroke Hypertension	–	7880	AH	Telephone interview

AC: anti-coagulants; AF: atrial fibrillation; AH: anti-hypertensives; AP: anti-platelets; AT: anti-thrombotics; BMQ: beliefs about medicines questionnaire; HS: haemorrhagic stroke; IS: ischaemic stroke; LLD: lipid-lowering drugs; MARS: medication adherence report scale; MMAS: Morisky-medication adherence scale.

#### Description of predictive factors for non-adherence

Two studies showed no difference in predictors within groups. One compared factors between rural and urban residence^35^ and the other compared patients living in different income quintiles.^28^ Factors related to non-adherence in the other 27 studies are classified below and detailed in the supplementary appendix.

##### Patient-related factors

Younger age at time of stroke was associated with reduced medication adherence in seven studies^9,10,18,24,26,33,34^ whereas younger age reported to associate with better adherence in five studies.^2,29,36,39,40^ Three studies reported that female sex predicted decreased adherence^2,29,32^ whereas one reported the opposite.^37^

Other patient-related factors included having concerns about medication, which associated with decreased adherence in four studies,^10,12,27,30^ or when patients perceived no benefit of treatment as reported in one study.^10^ On the other hand, when patients had positive beliefs about medication^23,25,37^ and indicated they were aware of the consequence of not taking prescribed medication,^23^ these factors were associated with enhanced adherence to medication.

##### Socioeconomic factors

Three studies indicated that having some sort of education^21,40^ or settled work status^18^ were associated with improved adherence. Four studies reported that the presence of patient carer or supporter also predicted better adherence.^2,23,25,29^ Two studies reported that living at care institution other than home was associated with worsened adherence.^2,39^

##### Therapy-related factors

Disease- or health-related factors that predicted non-adherence included disability,^1,9,18,29,37,39^ reduced cognition function,^10,23,25,37^ poor quality of life^2,11,18^ and low mood.^2,25^ Smoking^9,34^ and alcohol consumption^34,40^ were also predictors of medication non-adherence.

Existence of co-morbidities at the time of stroke associated with improved adherence to treatment. These included history of hypertension,^18,29,34^ diabetes,^2,18^ dyslipidaemia,^18,21,40^ coronary artery disease^18,40^ or myocardial infarction.^18,33^ Conversely, the absent history of atrial fibrillation was associated with better adherence.^2,18,29,36,40^

Prescribed regimen factors that predicted enhanced adherence included understanding of medication rationale,^1,18,23,30^ awareness of duration of treatment,^30^ knowledge of how to refill prescription,^18^ previous treatment by the same medication class,^2,38,40^ prescription and education at hospital discharge after the incident.^20^ Also, development of medication routine^23^ and use of compliance aid by patient.^1^

Medication regimen factors which associated with reduced adherence included cost of medication^9,19,22^ and number and frequency of prescribed drugs.^1,9,18,29^

##### Health system or caregiver-related factors

Caregiver-related factors included prescriber speciality (e.g. neurologist).^1^ Patient–caregiver relationship factors included language barrier, low trust, perceived discrimination, inadequate continuity of care^1^ and inadequate communication of information regarding prescribed regimen.^30^

Institution factors associated with better adherence included treating facility i.e. treated in stroke unit,^2,37^ treated in academic hospital^29^ and hospital size.^18^ Additionally, arrangement of medical insurance^11,24^ and accessible health care facility^2,12^ predicted enhanced adherence.

##### Stroke-related factors

Stroke-related factors that predicted non-adherence included delay from onset of symptoms to evaluation,^34^ symptoms of post-traumatic stress disorder (PTSD),^27,31^ more severe stroke,^33,36,39,40^ previous stroke incidence^2,9,37^ and time from stroke onset.^27^ Stroke subtype was another predictor of non-adherence e.g. ischaemic stroke versus Tia,^29^ cardio-embolic^36^ and haemorrhagic stroke.^2^ Nevertheless, factors like reduced cognition, disability and poor quality of life could also be stroke-related.

### Meta-analysis

**Figure 2.**
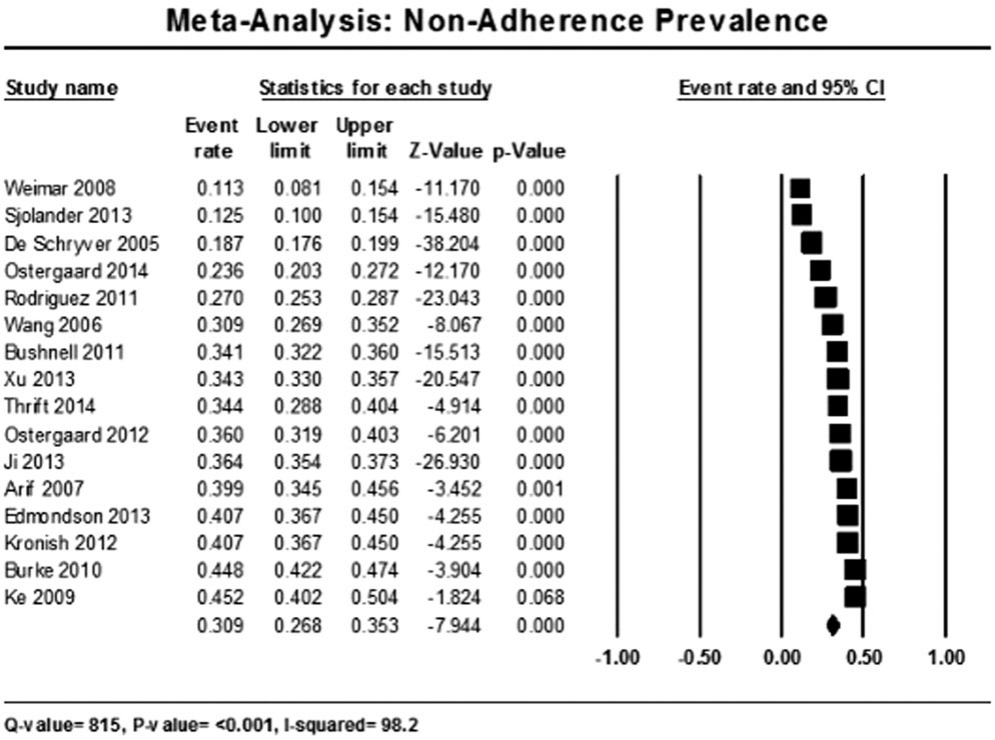
Meta-analysis of prevalence of non-adherence within included studies.

Sixteen studies were eligible for the meta-analysis of prevalence of non-adherence as they provided a measure of medication non-adherence rate.^1,11,20–22,26,27,29–31,33–35,37,39,40^ The rate of non-adherence was 30.9% (95% CI 26.8–35.3%) (Figure 2).

**Figure 3.**
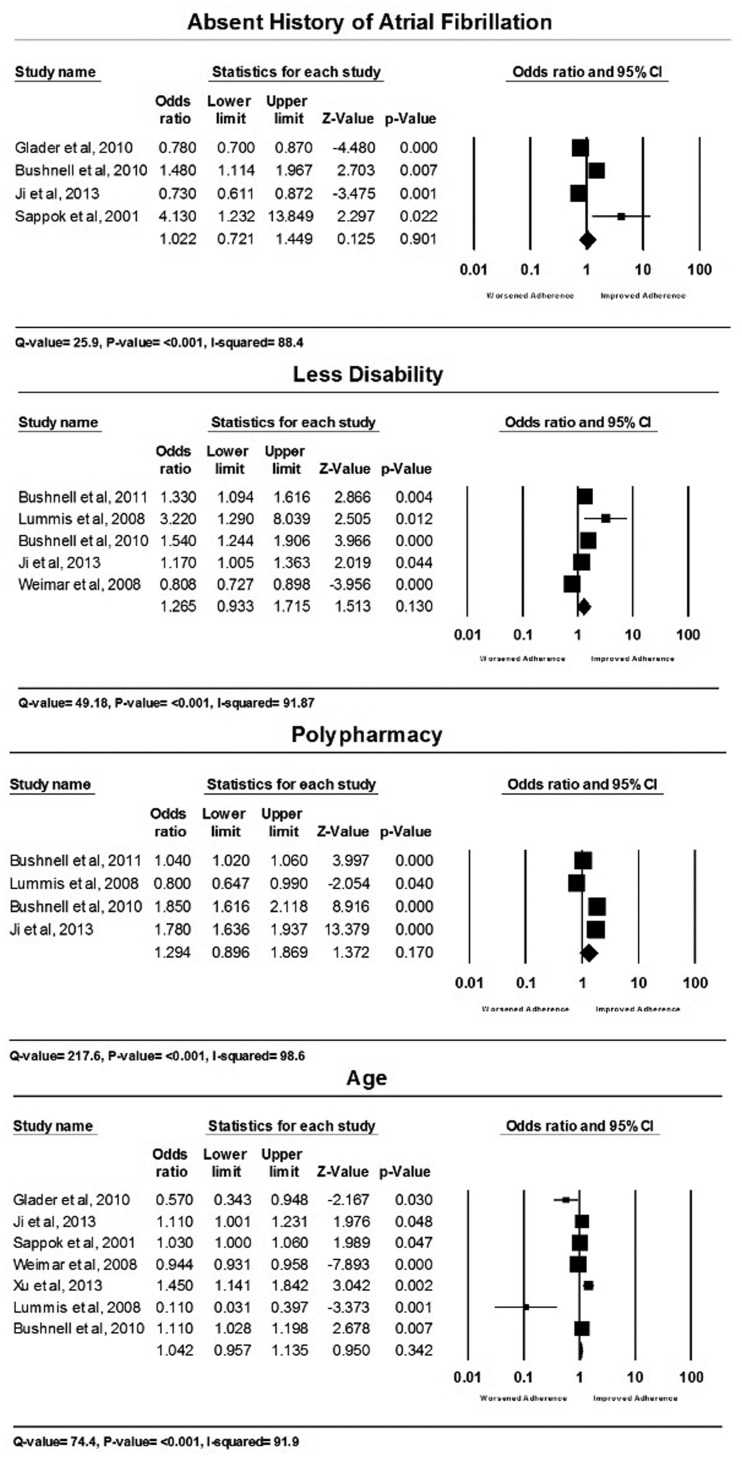
Meta-analyses of predictive factors.

For the meta-analysis of effect of factors on medication adherence, four factors were eligible which were: absent history of AF (4 studies^2,18,29,36^), disability (5 studies^1,9,18,29,39^), polypharmacy (4 studies^1,9,18,29^) and age of the patient (7 studies^2,9,18,29,36,39,40^). Meta-analyses of these factors showed that these factors did not significantly associate with medication adherence (no AF OR 1.02, 95% CI 0.72–1.5 (*p* = 0.9); disability OR 1.27, 95% CI 0.93–1.72 (*p* = 0.13); polypharmacy OR 1.29, 95% CI 0.9–1.9 (*p* = 0.17); age OR 1.04, 95% CI 0.96–1.14 (*p* = 0.34)). Forest plots for each factor analysis are available in Figure 3. There was considerable heterogeneity across all studies included in the meta-analyses (all *I*^2 ^> 88%).

## Discussion

In this review, we identified factors associated with adherence behaviour to secondary preventative medication after stroke or TIA. As stated by the WHO, patients alone used to be held responsible for non-adherence; however, it has been identified that other factors including the health care system or providers can also impact on non-adherence.^17^

Many factors associated with enhanced adherence to secondary preventative medication including positive beliefs about medication.^23,25,37^ This also included patients who encountered lower cost of medications^9,19,22^ or had medical insurance.^11,24^

Most of the published work focusses on patient and drug specific factors as determinants of adherence. The importance of institution or health care factors should not be neglected. Prescribing and educating patients on medication for secondary prevention before hospital discharge was linked to improved adherence.^20^ Numerous studies showed that in-hospital initiation of secondary preventative medication resulted in higher rates of adherence.^20,43,44^ This should include details on the purpose of treatment and regimen dosage.^1,18,23,30^ Also, patients should be ensured adequate continuity of care^1^ and access to health care after stroke.^2,12^ These simple measures could improve clinical outcomes.

Nonetheless, stroke patients with disability,^1,9,18,29,37,39^ reduced cognitive function,^10,23,25,37^ increased number of prescribed medication,^1,9,18,29^ concerns about treatment,^10,12,27,30^ history of stroke^2,9,37^ or more severe stroke event^33,36,39,40^ commonly showed reduced adherence to treatment.

Factors reported in this review were similar to those reported to correlate with adherence to medication in cardiovascular disease including coronary heart disease and acute coronary syndrome^45–48^ and to medications in general.^49,50^

Two patient-related factors were controversial in predicting adherence to secondary preventative medication, age at the time of stroke incident^2,9,10,18,24,29,33,34,36,39,40^ and sex of the patient.^2,29,32,37^ A study that assessed differences in prescribing secondary preventative drugs to stroke patients found significant differences where women were less likely to receive all recommended secondary preventative medication classes than men. However, younger patients were less likely to receive anti-platelet treatment.^51^ These factors are, however, non-reversible or amendable thus health care practitioners need to not hesitate with secondary prevention therapy if prescribing does not contrast with evidence-based recommendations.

In the meta-analysis of prevalence of non-adherence, we found non-adherence to be high with almost a third of stroke patients not receiving adequate secondary prevention. This clearly indicates importance for applying interventions that would improve adherence especially in the group vulnerable for non-adherence.

Despite the fact that none of the factors meta-analysed in this review showed significant association with medication adherence, caution should be taken not to interpret that association does not exist. This is explainable by the heterogeneity within included studies which was due to the considerable variation in subjects’ inclusion criteria, factors reported, medication classes, definition of adherence or compliance and the analysis used.

## Limitations

There were several limitations of this review. Available data are heterogeneous as a result of lack of universal reporting of medication adherence. In addition, there was no standardised scale to critically appraise type of included studies. Also, inclusion and exclusion specification could have influenced reporting predictors e.g. if a study excluded participants of specific age or population who are known to have a high risk of non-adherence.

## Implication for practice and future research

In this review, we aimed to identify factors correlated with adherence to secondary preventative medication after stroke. When clinicians are able to discuss barriers of adherence with their patients, they could ensure reducing the burden of treatment on their patients. It is also essential to identify reversible factors, e.g. misbeliefs or complex regimens, as these can be addressed. On the other hand, knowing factors that encourage stroke patients to adhere, clinicians would also be able to support stroke patients who are already adhering to maintain a good level of adherence. Researchers need to identify which interventions work best in supporting stroke patients to safely continue treatment with secondary preventative medication. Also, measures for detecting and tackling difficulties for medication administration after stroke need to be tested and implemented.

## Conclusion

Potential stroke patients with identified factors that predicted non-adherence require further attention, continuous encouragement and support with medication intake. Factors frequently reported to affect adherence included concerns about treatment regimen, increased disability, suffering severe stroke, polypharmacy and complex medication regimen. Focus should be more on reversible factors such as correcting misbeliefs about medication and providing convenient regimen. Stroke patients with disability or reduced cognition should be given additional care.

## Supplementary Material

Supplementary material
