# Supplementary material for: Predictive factors of non-adherence to secondary preventative medication after stroke or transient ischaemic attack: A systematic review and meta-analyses
Source: Eur Stroke J. 2016 May 5;1(2):65–75. doi: 10.1177/2396987316647187 (PMC5992740; doi:10.1177/2396987316647187)
Supplement: Supplementary material [file suppl-appendix.pdf]

Appendix 1: Search Terms

Medication Adherence

Adherence OR Compliance OR Concordance OR Persistence OR nonadherence OR non-adherence OR non-compliance OR noncompliance OR dosage adherence OR dosage compliance OR dose adherence OR dose compliance OR dosing adherence OR dosing compliance OR drug adherence OR drug compliance OR drug intake compliance OR drug regimen adherence OR drug regimen compliance OR medication adherence OR medication compliance OR medication intake adherence OR medication non adherence OR medication non compliance OR medication nonadherence OR medication non-adherence OR medication noncompliance OR medication non-compliance OR medication persistence OR nonadherence patient OR noncompliance patient OR patient adherence OR patient adherence OR patient compliance OR patient cooperation OR patient non adherence OR patient non compliance OR patient nonadherence OR patient non-adherence OR patient noncompliance OR patient non-compliance OR therapy adherence OR therapy compliance OR treatment adherence OR treatment compliance

Stroke

ACA Infarction OR acute cerebrovascular accident OR acute cerebrovascular lesion OR acute focal cerebral vasculopathy OR acute ischemic stroke OR acute stroke OR Anoxic Brain Damage OR Anoxic Encephalopathy OR Anoxic-Ischemic Encephalopathy OR Anterior Cerebral Artery Infarction OR Anterior Cerebral Artery Stroke OR Anterior Cerebral Artery Syndrome OR anterior cerebral circulation infarction OR anterior choroidal artery infarction OR anterior circulation brain infarction OR Anterior Circulation Transient Ischemic Attack OR apoplectic stroke OR apoplexia OR apoplexy OR Basal Ganglionic Hemorrhage OR Basilar Artery Insufficiency OR Basilar Artery Ischemia OR Basilar Artery Stenosis OR Basilar Insufficiency OR Benedict Syndrome OR brain accident OR Brain Anoxia OR Brain Anoxia-Ischemia OR brain arterial insufficiency OR brain attack OR brain blood flow disturbance OR brain circulation disorder OR Brain Hemorrhage OR Brain Hypoxia OR Brain Hypoxia-Ischemia OR brain infarct OR brain infarction OR brain insult OR brain ischaemia OR brain ischemic attack OR brain stem infarction OR Brain Stem Transient Ischemic Attack OR Brain TIA OR brain transient ischemic attack OR brain vascular accident OR brain venous infarction OR brainstem infarction OR Brainstem Stroke OR Brainstem Transient Ischemic Attack OR Carotid Circulation Transient Ischemic Attack OR cerebellar infarction OR cerebellum infarction OR Cerebral Anoxia OR Cerebral Anoxia-Ischemia OR cerebral apoplexia OR cerebral blood circulation disorder OR cerebral blood flow disorder OR Cerebral Brain Hemorrhage OR cerebral circulation disorder OR cerebral circulatory disorder OR Cerebral Hemorrhage OR Cerebral Hypoxia OR Cerebral Hypoxia-Ischemia OR cerebral infarct OR cerebral infarction OR cerebral insult OR cerebral ischaemia OR cerebral ischemia OR

Cerebral Parenchymal Hemorrhage OR cerebral stroke OR cerebral vascular accident OR cerebral vascular insufficiency OR cerebrovascular accident OR cerebrovascular apoplexy OR cerebrovascular arrest OR cerebrovascular circulation disorder OR cerebrovascular failure OR cerebrovascular infarction OR cerebrovascular injury OR cerebrovascular insufficiency OR cerebrovascular insult OR cerebrovascular ischaemia OR cerebrovascular ischemia OR cerebrovascular stroke OR cerebrovascular trauma OR Cerebrum Hemorrhage OR cerebrum vascular accident OR chronic ischemic stroke OR circulatory epilepsy OR Claude Syndrome OR cortical infarction OR Crescendo Transient Ischemic Attacks OR cryptogenic stroke OR CVA OR cvas OR Foville Syndrome OR Hematoma, Basal Ganglia OR hemisphere infarct OR hemisphere infarction OR hemispheric infarct OR hemispheric infarction OR Hemorrhage, Basal Ganglia OR Heubner Artery Infarction OR Heubner's Artery Infarction OR Hypoxic Brain Damage OR Hypoxic Encephalopathy OR Hypoxic-Ischemic Encephalopathy OR Infarction, Anterior Cerebral Artery Circulation OR Infarction Anterior Cerebral Artery Distribution OR infarctions lacunar OR Intracerebral Hemorrhage OR Intracranial Hemorrhage OR ischaemic attack OR ischaemic brain disease OR ischaemic seizure OR ischaemic stroke OR ischemia cerebri OR ischemic attack OR ischemic brain disease OR ischemic cerebral attack OR ischemic encephalopathy OR ischemic seizure OR ischemic stroke OR lacunar infarct OR lacunar infarction OR lacunar stroke OR lacunar syndrome OR left hemisphere infarction cerebral OR Left Middle Cerebral Artery Infarction OR MCA Infarction OR Middle Cerebral Artery Circulation Infarction OR Middle Cerebral Artery Embolic Infarction OR Middle Cerebral Artery Embolus OR Middle Cerebral Artery Infarction OR Middle Cerebral Artery Occlusion OR Middle Cerebral Artery Stroke OR Middle Cerebral Artery Syndrome OR Middle Cerebral Artery Thrombosis OR Middle Cerebral Artery Thrombotic Infarction OR Millard-Gublar Syndrome OR neural ischemia OR PCA Infarction OR Perinatal Subarachnoid Hemorrhage OR Posterior Cerebral Artery Embolic Infarction OR Posterior Cerebral Artery Infarction OR Posterior Cerebral Artery Stroke OR Posterior Cerebral Artery Syndrome OR Posterior Cerebral Artery Thrombotic Infarction OR posterior choroidal artery infarction OR posterior circulation brain infarction OR Posterior Circulation Transient Ischemic Attack OR Posterior Fossa Hemorrhage OR right hemisphere cerebral infarction OR Right Middle Cerebral Artery Infarction OR SAH OR silent brain infarction OR stroke OR subarachnoid hemorrhage OR Subarachnoid Hemorrhage Aneurysmal OR Subarachnoid Hemorrhage Intracranial OR Subarachnoid Hemorrhage Spontaneous OR subcortical infarction OR TIA OR Top of the Basilar Syndrome OR transient brain ischemia OR Transient Brain Stem Ischemia OR Transient Brainstem Ischemia OR Transient Cerebral Ischemia OR Transient Ischemic Attack OR transient ischemic seizure OR Vertebral Artery Insufficiency OR Vertebral Artery Ischemia OR Vertebral Artery Stenosis OR Vertebrobasilar Circulation Transient Ischemic Attack OR Vertebrobasilar Dolichoectasia OR Vertebro-Basilar Insufficiency OR Vertebrobasilar Ischemia OR Vertebro-Basilar Ischemia OR Weber Syndrome.

Appendix 2: PRISMA Checklist

Section/topic	#	Checklist item	Reported on page #
**TITLE**			
Title	1	Identify the report as a systematic review, meta-analysis, or both.	1
**ABSTRACT**			
Structured summary	2	Provide a structured summary including, as applicable: background; objectives; data sources; study eligibility criteria, participants, and interventions; study appraisal and synthesis methods; results; limitations; conclusions and implications of key findings; systematic review registration number.	2 & 3
**INTRODUCTION**			
Rationale	3	Describe the rationale for the review in the context of what is already known.	4
Objectives	4	Provide an explicit statement of questions being addressed with reference to participants, interventions, comparisons, outcomes, and study design (PICOS).	4
**METHODS**			
Protocol and registration	5	Indicate if a review protocol exists, if and where it can be accessed (e.g., Web address), and, if available, provide registration information including registration number.	5
Eligibility criteria	6	Specify study characteristics (e.g., PICOS, length of follow-up) and report characteristics (e.g., years considered, language, publication status) used as criteria for eligibility, giving rationale.	5
Information sources	7	Describe all information sources (e.g., databases with dates of coverage, contact with study authors to identify additional studies) in the search and date last searched.	5
Search	8	Present full electronic search strategy for at least one database, including any limits used, such that it could be repeated.	5 & Appendix
Study selection	9	State the process for selecting studies (i.e., screening, eligibility, included in systematic review, and, if applicable, included in the meta-analysis).	5 & 6
Data collection process	10	Describe method of data extraction from reports (e.g., piloted forms, independently, in duplicate) and any processes for obtaining and confirming data from investigators.	6
**Section/topic**	**#**	**Checklist item**	**Reported on page #**

Data items	11	List and define all variables for which data were sought (e.g., PICOS, funding sources) and any assumptions and simplifications made.	6
Risk of bias in individual studies	12	Describe methods used for assessing risk of bias of individual studies (including specification of whether this was done at the study or outcome level), and how this information is to be used in any data synthesis.	Appendix
Summary measures	13	State the principal summary measures (e.g., risk ratio, difference in means).	-
Synthesis of results	14	Describe the methods of handling data and combining results of studies, if done, including measures of consistency (e.g., I^2) for each meta-analysis.	6
Risk of bias across studies	15	Specify any assessment of risk of bias that may affect the cumulative evidence (e.g., publication bias, selective reporting within studies).	6
Additional analyses	16	Describe methods of additional analyses (e.g., sensitivity or subgroup analyses, meta-regression), if done, indicating which were pre-specified.	7
**RESULTS**			
Study selection	17	Give numbers of studies screened, assessed for eligibility, and included in the review, with reasons for exclusions at each stage, ideally with a flow diagram.	7 & figure 1
Study characteristics	18	For each study, present characteristics for which data were extracted (e.g., study size, PICOS, follow-up period) and provide the citations.	8 – 9 & table 1
Risk of bias within studies	19	Present data on risk of bias of each study and, if available, any outcome level assessment (see item 12).	7 & appendix
Results of individual studies	20	For all outcomes considered (benefits or harms), present, for each study: (a) simple summary data for each intervention group (b) effect estimates and confidence intervals, ideally with a forest plot.	8 – 11 & appendix
Synthesis of results	21	Present results of each meta-analysis done, including confidence intervals and measures of consistency.	11
Risk of bias across studies	22	Present results of any assessment of risk of bias across studies (see Item 15).	7 & appendix
Additional analysis	23	Give results of additional analyses, if done (e.g., sensitivity or subgroup analyses, meta-regression [see Item 16]).	N/A
**DISCUSSION**			
**Section/topic**	**#**	**Checklist item**	**Reported on page #**
Summary of evidence	24	Summarize the main findings including the strength of evidence for each main outcome; consider their relevance to key groups (e.g., healthcare providers, users, and policy makers).	12 & 13

Limitations	25	Discuss limitations at study and outcome level (e.g., risk of bias), and at review-level (e.g., incomplete retrieval of identified research, reporting bias).	13
Conclusions	26	Provide a general interpretation of the results in the context of other evidence, and implications for future research.	14
**FUNDING**			
Funding	27	Describe sources of funding for the systematic review and other support (e.g., supply of data); role of funders for the systematic review.	15

Table 1: Significance of Predictive Factors										
**Study author, year & direction of effect**	**Analysis used**	**Medication classes studied**	**N**	**Overall non-adherence (%)**	**Predictive factor**	**Significance**				
						***p*-Value**	**OR**	**HR**	**95% CI Lower**	**95% CI Upper**
Arif et al, 2007<br>Improved adherence	Multivariable analysis with forward conditional logistic regression	Antiplatelets<br>Antihypertensives<br>Lipid-lowering drugs	298	119 (40)	School education (minimum)<br>Dyslipidaemia (not with other co-morbidities)	0.004<br>0.018	2.1<br>0.5	---<br>---	1.3<br>0.3	3.5<br>0.9
Burke et al, 2010<br>Worsened adherence	Cox proportional hazard	Antiplatelets	1413	633 (44.8)	Higher medication co-payment > $40 (relative to co-payment of ≤ $20)	0.0042	---	1.32	1.091	1.596
Bushnell et al, 2010<br>Improved adherence	Multivariable logistic regression analyses	Antiplatelets<br>Warfarin<br>Antihypertensives<br>Lipid-lowering drugs<br>Diabetes medications	2598	N/A	Fewer prescribed medication classes	<0.001	1.85	---	1.61	2.11
					Increasing age	0.005	1.11		1.03	1.20
					No history of atrial fibrillation	0.007	1.48		1.11	1.96
					History of hypertension	<0.001	1.47		1.17	1.84
					History of diabetes mellitus	0.008	1.39		1.09	1.78
					History of dyslipidaemia	<0.001	1.46		1.20	1.79
					History of CAD/prior MI	0.02	1.32		1.05	1.68
					Less severe stroke disability (mRS < 3)	<0.001	1.54		1.24	1.90

					Working status:					
					Home not by choice vs home by choice	0.06	1.61		1.08	2.42
					Home not by choice vs working	---	1.47		1.00	2.15
					Understanding why medications are prescribed	0.006	1.81		1.19	2.76
					Understanding how to refill medication	0.03	1.64		1.04	2.58
					Increased quality of life (EuroQol-5D, per 10% increase)	0.009	2.33		1.24	4.38
					Financial hardship	0.002	1.38		1.12	1.69
					Hospital size (per 100-bed increase)	0.052	1.04		1.02	1.09
Bushnell et al, 2011 Improved adherence & persistence	Multivariable logistic regression	Warfarin Antiplatelets Antihypertensives Lipid-lowering drugs Diabetic drugs	2457	837 (34.1)	Fewer medications at discharge	0.001	1.04	---	1.02	1.06
					Adequate income	0.003	1.30		1.10	1.55
					Appointment with a primary care provider	0.027	1.47		1.05	2.07
					Appointment with neurologist	0.023	1.20		1.03	1.41
					Lower mRS score (≤3)	0.006	1.33		1.09	1.61

					Understanding of medications rational & side effects<br>Used compliance aid (pillbox)	0.003<br><br>0.001	1.43<br><br>1.89		1.13<br><br>1.48	1.81<br><br>2.41
Chambers et al, 2011 (subgroup analysis from O'Carroll *et al*, 2008)<br>Improved adherence	Qualitative analysis	Not specified	26	13 (50)	Medication routine<br>Positive beliefs about medication and treatment<br>Knowledge about the medication rationale or intentions<br>Support from family and health professionals<br>Realistic understanding of the consequences of non-adherence<br>Not forgetting to take medication	---<br><0.05<br><br><0.05	---<br>Mean differe nce = −4.4<br>Mean differe nce % = 24.6	---	---<br>−7.6<br><br>0.5	---<br>−1.2<br><br>48.7
Choi-Kwon et al, 2005<br>Worsened adherence	Multiple logistic regression	Antihypertensives	256	N/A	Early-onset patients (younger age)<br>Lack of insurance	<0.001<br><br><0.05	---<br><br>0.096		---<br><br>0.008	---<br><br>0.976

Coetzee et al, 2008 Worsened adherence	Regression analysis	All preventative classes plus other classes	26	N/A	Cognitive dysfunction	<0.001	---	---	---	---
					Emotional dysfunction	<0.001				
					Misbeliefs about medication	<0.001				
					Less support with medication intake at home	<0.001				
De Schryver et al, 2005	Cox proportional hazard	Aspirin<br>Oral anticoagulants	4447	832 (18.7)	<u>Aspirin (worst):</u>	---	---			
					Age ≥ 65 years			1.31	1.04	1.64
					Higher doses (300 mg instead of 30 mg)			1.30	1.03	1.62
					<u>Aspirin (better):</u>					
					Diastolic blood pressure of ≥ 90 mmHg			0.74	0.59	0.93
					Dizziness			0.54	0.33	0.89
					<u>Oral anti-coagulant:</u>					
					None identified					
Edmondson et al, 2013 Worsened adherence	Multivariable logistic regression analysis	Antithrombotics<br>Antihypertensives<br>Lipid-lowering drugs<br>Diabetes medications	535	218 (40.8)	Symptoms of PTSD	---	1.02	---	1.00	1.05
					Concerns about medications		1.17		1.10	1.25
					Years since last stroke		1.17		1.02	1.34

Glader et al, 2010<br>Worst adherence	Multiple logistic regression with stepwise backward variable removal (*P* > 0.10 as removal criterion)	Anti-hypertensives<br>Statins<br>Antiplatelet drugs<br>Warfarin	21077	N/A	<u>Anti-hypertensives:</u>			---		
					Female gender	<0.001	0.81		0.75	0.89
					Haemorrhagic stroke	0.004	1.24		1.07	1.4
					History of diabetes	0.01	1.17		1.06	1.30
					History of AF	<0.001	1.24		1.12	1.38
					Previous treatment	<0.001	2.07		1.89	2.27
					Institutional living	<0.001	1.47		1.27	1.70
					Low mood	0.001	0.88		0.79	0.98
					Poor self-perceived health	0.02	0.86		0.76	0.98
					Lack of support	0.001	1.13		1.02	1.25
					<u>Statins:</u>					
					Recurrent stroke	<0.001	0.77		0.68	0.88
					No acute treatment in stroke unit	0.007	1.25		1.06	1.46
					Previous treatment	NS?	1.28		1.15	1.42
					Institutional living	0.001	1.64		1.32	2.04
					Poor self-perceived health	<0.001	0.69		0.59	0.80
					No outpatient follow-up	0.05	1.04		0.93	1.17
					<u>Antiplatelet drugs:</u>					
					Female gender	0.04	0.92		0.85	0.99

					Advanced age (>85)	<0.001	1.37		1.20	1.57
					Absent history of AF	<0.001	0.78		0.70	0.87
					Institutional living	<0.001	1.78		1.55	2.05
					Low mood	0.04	0.92		0.83	1.02
					Poor self-perceived health	<0.001	0.79		0.70	0.89
					Warfarin:					
					Advanced age (>85)	0.01	0.57		0.34	0.94
					Recurrent stroke	0.04	0.70		0.53	0.93
					Institutional living	0.004	0.45		0.27	0.76
					Lack of support	0.05	0.98		0.76	1.26
Huang et al, 2013	Multivariable logistic regression	Antihypertensives<br>Antithrombotics<br>Lipid-lowering drugs	11050	N/A	There was no difference across income quintiles in the use of post discharge medications	---	---	---	---	---
Ji et al, 2013<br>Improved adherence	Univariate and multivariate logistic regression (stepwise backward method with likelihood ratio test)	Antiplatelets<br>Warfarin<br>Antihypertensives<br>Statins<br>Diabetic agents	9998	3635 (36.4)	Univariate (+Multivariate):					
					Younger age (≤75)	0.05	1.11	---	1.00	1.23
					Male gender	0.003	1.14		1.05	1.24
					Race (Hans)	0.003	1.40		1.23	1.75
					Married	0.05	1.15		1.00	1.32
					History of hypertension	0.05	1.09		1.00	1.19
					Absent history of diabetes	0.01	0.88		0.80	0.98

					Absent history of atrial fibrillation<br>Pre-stroke independence<br>Acute ischaemic stroke vs TIA<br>Treated in an academic hospital<br>Longer hospital stay<br>Fewer number of medications	0.01<br>0.04<br>0.005<br>0.003<br>0.005<br>0.001	0.73<br>1.17<br>1.22<br>1.13<br>1.01<br>1.78		0.61<br>1.01<br>1.06<br>1.04<br>1.00<br>1.63	0.87<br>1.37<br>1.39<br>1.23<br>1.01<br>1.93
Ke et al, 2009 Worsened adherence	Only reported count & percentage	Aspirin	367	166 (45.2)	Concerns about side effects<br>Inadequate communication with prescriber	---	---	---	---	---
Kronish et al, 2012 Worsened adherence	Logistic regression	Not specified	535	218 (40.8)	PTSD (post-traumatic stress disorder)	---	Relative risk: 2.90	---	1.92	4.42
Kronish et al, 2013 Worsened adherence	Logistic regression	Not specified	600	242 (40.3)	Increased concerns about medications<br>Low trust in doctor<br>Language barrier<br>Perceived discrimination<br>Difficulty accessing healthcare	<0.001<br>0.001<br>0.02<br><0.001<br>0.002	---	---	---	---

					Inadequate continuity of care	0.05				
					<u>Adjusted model:</u>					
					Increased concerns about medication	<0.001	5.02		2.76	9.11
					Perceived discrimination	0.008	1.85		1.18	2.90
Levine et al, 2013<br>Worsened adherence	Multivariable logistic regression analyses	Not specified	8673	N/A	Cost-related non-adherence (Self-reported):		---	---	---	---
					Aged 45 – 64	0.01				
					Aged ≥ 65	0.21				
Lopes et al, 2011<br>Improved adherence	Multivariate logistic regression	Warfarin	291	N/A	Male gender	0.01	2.27	---	1.22	4.35
Lummis et al, 2008	Multivariable analysis (for each medication class separately)	Anti-thrombotics<br>Anti-hypertensives<br>Lipid-lowering drugs<br>Diabetes medications	420	N/A	<u>Anti-hypertensives:</u>					
					Age between (65 – 79)	---	0.11	---	0.03	0.39
					Number of medication prescribed		0.80		0.65	0.995
					Monthly drug cost:					
					< $90		7.05		1.43	34.81
					$90 - $199		5.76		1.27	26.18
					<u>Anti-thrombotics:</u>					
					Age between (65 – 79)		0.23		0.06	0.81

					Disability before stroke<br>Disability after stroke<br>Lipid-lowering drugs:<br>Smoking<br>History of previous stroke		7.01<br>3.22<br><br>4.35<br>0.13		1.66<br>1.29<br><br>1.44<br>0.02	29.58<br>8.04<br><br>13.13<br>1.004
O'Carroll et al, 2011<br>Worsened adherence	Multivariable- Hierarchical linear regression	Antihypertensives<br>Aspirin<br>Statins	180	N/A	Time 1:<br>Younger age<br>Concerns about medications<br>Reduced cognitive functioning (MMSE score)<br>Low perceived benefit of medication.<br>Time 2:<br>Younger age<br>Concerns about medications<br>Low perceived benefit of medication	 <0.001<br><0.001<br>0.01<br>0.043<br><br>0.004<br>0.007<br>0.001	 0.371<br>0.355<br>0.201<br>0.159<br><br>0.241<br>0.254<br>0.273	---	---	---
Østergaard et al, 2012<br>Worsened	Univariate and multivariate analysis (cox regression)	Antiplatelets	503	181 (36)	Univariate:<br>Younger age<br>Severe stroke	 0.005<br><0.001	 ---<br>---	 ---<br>---	 ---<br>---	 ---<br>---

persistence					History of MI<br>Multivariate:<br>Stroke severity	0.03<br><br>---	---<br><br>---	---<br><br>0.47	---<br><br>0.30	---<br><br>0.74
Østergaard et al, 2014<br>Worsened persistence	Cox proportional hazards model (univariate) then unconditional logistic regression for multivariate	Antiplatelets	594	140 (23.6)	Univariate:<br>Younger age (<55 years)<br>Current smoking status<br>High alcohol intake<br>History of hypertension<br>Multivariate:<br>Younger age (<54)<br>Delay between TIA onset and neurological evaluation (7+ days)	 <0.001<br>0.05<br>0.03<br>0.002	---	---<br><br><br><br><br><br>1.9<br>2.0	---<br><br><br><br><br><br>1.3<br>1.0	---<br><br><br><br><br><br>2.8<br>4.1
Rodriguez et al, 2011	Multivariate logistic model	Antiplatelets<br>Warfarin<br>Antihypertensives<br>Lipid-lowering agents<br>Diabetic agents	2720	734 (27)	No differences between rural & urban residence in medication persistence post discharge	---	---	---	---	---
Sappok et al, 2001<br>Worsened	Stepwise backward logistic regression	Antithrombotics	386	N/A	Higher age<br>Stroke Severity on admission	---	1.03<br>1.09	---	1.00<br>1.00	1.06<br>1.20

adherence					Cardio-embolic cause		4.13		1.23	13.83
Sjölander et al, 2012<br>Improved adherence	Multiple log-linear Poisson regression	Antihypertensives	18349	N/A	Previous treatment with antihypertensive drug:<br>Men (Prevalence ratio, PR)<br>Women (PR)	---	 <br>1.15<br>1.16	---	 <br>1.06<br>1.06	 <br>1.25<br>1.27
Sjölander et al, 2013<br>Worsened adherence	Multivariable logistic regression models	Not specified	578	72 (12.5)	Negative beliefs about medicines<br>Male gender<br>Not treated in stroke units<br>Dependent on the help and support from relatives<br>History of stroke<br>Self-reported memory difficulties	 <br>0.042<br>0.016<br>0.015<br><br>0.01<br>0.014	1.12	---	1.05	1.21
Thrift et al, 2014<br>Improved adherence	Logistic regression	Antihypertensives<br>Antithrombotics<br>Statins	256	88 (34.4)	Prescription of medications at hospital discharge	---	2.62	---	1.19	5.77
Wang et al, 2006.	Univariate odds ratio (OR) values	Antithrombotics	472	146 (19.7)	Improved adherence:<br>Medical insurance or free medical care	 ---	 1.624	 ---	 1.004	 2.626

					<u>Worst adherence:</u>					
					Non-aspirin based regimen	---	0.465		0.238	0.908
					Poor quality of daily life (BI)	0.01	---		---	---
Weimar et al, 2008 Worsened adherence	Stepwise logistic regression analysis	Oral anticoagulants	293	33 (11.3)	Higher age	<0.001	0.944	---	0.930	0.957
					Greater baseline stroke severity on the NIHSS	<0.001	0.943		0.918	0.969
					Discharge to another institution than home	0.003	1.599		1.175	2.176
					Higher disability on the mRS at discharge	<0.001	0.808		0.727	0.898
Xu et al, 2013	Multivariate logistic regression	Antihypertensives	4458	1531 (34.3)	<u>Worst adherence:</u>					
					Older age (>/= 76)	---	1.45	---	1.14	1.84
					History of AF		1.25		1.04	1.15
					History of drinking		1.19		1.01	1.41
					Higher NIHSS (≥15)		1.41		1.14	1.74
					<u>Improved adherence:</u>					
					Higher level of education		0.75		0.66	0.85
					History of hyperlipidaemia		0.90		0.81	0.99
					History of CAD		0.87		0.76	0.99
					Previous treatment with anti-		0.69		0.62	0.76

					hypertensive					
AF: Atrial fibrillation, AH: Antihypertensive drugs, AT: Anti-thrombotic drugs, BI: Barthel Index, CAD: Coronary artery disease, LL: Lipid-lowering drugs, MI: Myocardial infarction, mRS: modified Rankin Scale, N/A: Not available, NIHSS: National Institute of Health Stroke Scale, TIA: Transient ischaemic attack										

Table 2: Classification of Predictive Factors of Adherence to Secondary Preventative Medication after Stroke	
Factors Predicted Better Adherence	
**Factor**	**Studies reported**
Education	Arif et al, 2007<br>Xu et al, 2013
Working status	Bushnell et al, 2010
Presence of carer or supporter	Glader et al, 2010<br>Chambers et al, 2011<br>Coetzee et al, 2008<br>Ji et al, 2013
Positive beliefs about medication	Chambers et al, 2011<br>Coetzee et al, 2008<br>Sjölander et al, 2013
Awareness of consequence of not taking medication	Chambers et al, 2011
History of hypertension	Bushnell et al, 2010<br>Ji et al, 2013<br>Østergaard et al, 2014
History of diabetes	Glader et al, 2010<br>Bushnell et al, 2010
History of dyslipidaemia	Bushnell et al, 2010<br>Arif et al, 2007<br>Xu et al, 2013
History of coronary artery disease	Bushnell et al, 2010<br>Xu et al, 2013
History of myocardial infarction	Bushnell et al, 2010<br>Østergaard et al, 2012

Absent history of atrial fibrillation	Glader et al, 2010<br>Bushnell et al, 2010<br>Ji et al, 2013<br>Sappok et al, 2001<br>Xu et al, 2013
Understanding of medication rationale	Bushnell et al, 2011<br>Bushnell et al, 2010<br>Chambers et al, 2011<br>Ke et al, 2009
Awareness of duration of treatment	Ke et al, 2009
Knowledge of how to refill prescription	Bushnell et al, 2010
Previous treatment by the same medication class	Glader et al, 2010<br>Sjölander et al, 2012<br>Xu et al, 2013
Prescription and education at discharge	Thrift et al, 2014
Development of medication routine	Chambers et al, 2011
Use of compliance aid	Bushnell et al, 2011
Prescriber speciality (e.g. neurologist)	Bushnell et al, 2011
Treatment in stroke unit	Glader et al, 2010<br>Sjölander et al, 2013
Treatment in academic hospital	Ji et al, 2013
Hospital size	Bushnell et al, 2010
Medical insurance	Wang et al, 2006<br>Choi-Kwon et al, 2005
Accessible healthcare facility	Glader et al, 2010<br>Kronish et al, 2013

Factors Predicted Worst Adherence	
Having concerns about medication	O'Carroll et al, 2011<br>Kronish et al, 2013<br>Edmondson et al, 2013<br>Ke et al, 2009
No perceived benefit of treatment	O'Carroll et al, 2011
Living at care institution other than home	Glader et al, 2010<br>Weimar et al, 2008
Disability	Bushnell et al, 2011<br>Lummis et al, 2008<br>Bushnell et al, 2010<br>Ji et al, 2013<br>Sjölander et al, 2013<br>Weimar et al, 2008
Reduced cognition function	O'Carroll et al, 2011<br>Chambers et al, 2011<br>Coetzee et al, 2008<br>Sjölander et al, 2013
Poor quality of life	Glader et al, 2010<br>Wang et al, 2006<br>Bushnell et al, 2010
Low mood	Glader et al, 2010<br>Coetzee et al, 2008
Smoking	Lummis et al, 2008<br>Østergaard et al, 2014
Alcohol consumption	Østergaard et al, 2014<br>Xu et al, 2013

Cost of medication	Lummis et al, 2008<br>Levine et al, 2013<br>Burke et al, 2010
Number and frequency of prescribed drugs	Bushnell et al, 2011<br>Lummis et al, 2008<br>Bushnell et al, 2010<br>Ji et al, 2013
Higher dose (of aspirin)	De Schryver et al, 2005
Non-aspirin based regiment (for Anti-platelet drugs)	Wang et al, 2006
Language barrier	Bushnell et al, 2011
Low trust in healthcare provider	Bushnell et al, 2011
Perceived discrimination (e.g. minorities)	Bushnell et al, 2011
Inadequate continuity of care	Bushnell et al, 2011
Inadequate communication of information regarding prescribed regimen	Ke et al, 2009
delay from onset of symptoms to evaluation	Østergaard et al, 2014
Post-traumatic stress disorder (PTSD)	Edmondson et al, 2013<br>Kronish et al, 2012
More severe stroke	Østergaard et al, 2012<br>Sappok et al, 2001<br>Weimar et al, 2008<br>Xu et al, 2013
Previous stroke incidence	Glader et al, 2010<br>Lummis et al, 2008<br>Sjölander et al, 2013
Time from stroke onset	Edmondson et al, 2013
Ischaemic stroke vs. TIA	Ji et al, 2013

Cardio-embolic cause	Sappok et al, 2001
Haemorrhagic stroke	Glader et al, 2010
Contradictory Factors	
Age: Younger age better adherence	Glader et al, 2010<br>Ji et al, 2013<br>Sappok et al, 2001<br>Weimar et al, 2008<br>Xu et al, 2013
Age: Younger age worst adherence	Lummis et al, 2008<br>O'Carroll et al, 2011<br>Bushnell et al, 2010<br>Choi-Kwon et al, 2005<br>De Schryver et al, 2005<br>Østergaard et al, 2012<br>Østergaard et al, 2014
Gender: Women adhere better	Sjölander et al, 2013
Gender: Women adhere worst	Glader et al, 2010<br>Ji et al, 2013<br>Lopes et al, 2011

Table 3: Quality Assessment of Included Studies

Author, year	Selection Bias			Performance Bias / Detection Bias	Attrition Bias	Reporting Bias	Confounders	Overall Quality
	Clear Inclusion Criteria?	Representative Sample?	Control Population Well Defined?	Blinding of outcome assessors?	Incomplete outcome data addressed?	Free of selective outcome reporting?	Appropriate consideration of potential confounders?	
Arif et al, 2007	Yes<br>No<br>Unclear	Yes<br>No<br>Unclear	Yes<br>No<br>NA	Yes<br>No<br>Unclear	Yes<br>No<br>Unclear	Yes<br>No<br>Unclear	Yes<br>No<br>Unclear	**Low**<br>**High**
Burke et al, 2010	Yes<br>No<br>Unclear	Yes<br>No<br>Unclear	Yes<br>No<br>NA	Yes<br>No<br>Unclear	Yes<br>No<br>Unclear	Yes<br>No<br>Unclear	Yes<br>No<br>Unclear	**Low**<br>**High**
Bushnell et al, 2010	Yes<br>No<br>Unclear	Yes<br>No<br>Unclear	Yes<br>No<br>NA	Yes<br>No<br>Unclear	Yes<br>No<br>Unclear	Yes<br>No<br>Unclear	Yes<br>No<br>Unclear	**Low**<br>**High**
Bushnell et al, 2011	Yes<br>No<br>Unclear	Yes<br>No<br>Unclear	Yes<br>No<br>NA	Yes<br>No<br>Unclear	Yes<br>No<br>Unclear	Yes<br>No<br>Unclear	Yes<br>No<br>Unclear	**Low**<br>**High**
Chambers et al, 2011	Yes<br>No<br>Unclear	Yes<br>No<br>Unclear	Yes<br>No<br>NA	Yes<br>No<br>Unclear	Yes<br>No<br>Unclear	Yes<br>No<br>Unclear	Yes<br>No<br>Unclear	**Low**<br>**High**
Choi-Kwon et al, 2005	Yes<br>No<br>Unclear	Yes<br>No<br>Unclear	Yes<br>No<br>NA	Yes<br>No<br>Unclear	Yes<br>No<br>Unclear	Yes<br>No<br>Unclear	Yes<br>No<br>Unclear	**Low**<br>**High**
Coetzee et al, 2008	Yes	Yes	Yes	Yes	Yes	Yes	Yes	**Low**

	No<br>Unclear	No<br>Unclear	No<br>NA	No<br>Unclear	No<br>Unclear	No<br>Unclear	No<br>Unclear	High
De Schryver et al, 2005	Yes<br>No<br>Unclear	Yes<br>No<br>Unclear	Yes<br>No<br>NA	Yes<br>No<br>Unclear	Yes<br>No<br>Unclear	Yes<br>No<br>Unclear	Yes<br>No<br>Unclear	**Low**<br>**High**
Edmondson et al, 2013	Yes<br>No<br>Unclear	Yes<br>No<br>Unclear	Yes<br>No<br>NA	Yes<br>No<br>Unclear	Yes<br>No<br>Unclear	Yes<br>No<br>Unclear	Yes<br>No<br>Unclear	**Low**<br>**High**
Glader et al, 2010	Yes<br>No<br>Unclear	Yes<br>No<br>Unclear	Yes<br>No<br>NA	Yes<br>No<br>Unclear	Yes<br>No<br>Unclear	Yes<br>No<br>Unclear	Yes<br>No<br>Unclear	**Low**<br>**High**
Huang et al, 2013	Yes<br>No<br>Unclear	Yes<br>No<br>Unclear	Yes<br>No<br>NA	Yes<br>No<br>Unclear	Yes<br>No<br>Unclear	Yes<br>No<br>Unclear	Yes<br>No<br>Unclear	**Low**<br>**High**
Ji et al, 2013	Yes<br>No<br>Unclear	Yes<br>No<br>Unclear	Yes<br>No<br>NA	Yes<br>No<br>Unclear	Yes<br>No<br>Unclear	Yes<br>No<br>Unclear	Yes<br>No<br>Unclear	**Low**<br>**High**
Ke et al, 2009	Yes<br>No<br>Unclear	Yes<br>No<br>Unclear	Yes<br>No<br>NA	Yes<br>No<br>Unclear	Yes<br>No<br>Unclear	Yes<br>No<br>Unclear	Yes<br>No<br>Unclear	**Low**<br>**High**
Kronish et al, 2012	Yes<br>No<br>Unclear	Yes<br>No<br>Unclear	Yes<br>No<br>NA	Yes<br>No<br>Unclear	Yes<br>No<br>Unclear	Yes<br>No<br>Unclear	Yes<br>No<br>Unclear	**Low**<br>**High**
Kronish et al, 2013	Yes<br>No<br>Unclear	Yes<br>No<br>Unclear	Yes<br>No<br>NA	Yes<br>No<br>Unclear	Yes<br>No<br>Unclear	Yes<br>No<br>Unclear	Yes<br>No<br>Unclear	**Low**<br>**High**
Levine et al, 2013	Yes	Yes	Yes	Yes	Yes	Yes	Yes	**Low**

	No<br>Unclear	No<br>Unclear	No<br>NA	No<br>Unclear	No<br>Unclear	No<br>Unclear	No<br>Unclear	**High**
Lopes et al, 2011	Yes<br>No<br>Unclear	Yes<br>No<br>Unclear	Yes<br>No<br>NA	Yes<br>No<br>Unclear	Yes<br>No<br>Unclear	Yes<br>No<br>Unclear	Yes<br>No<br>Unclear	**Low**<br>**High**
Lummis et al, 2008	Yes<br>No<br>Unclear	Yes<br>No<br>Unclear	Yes<br>No<br>NA	Yes<br>No<br>Unclear	Yes<br>No<br>Unclear	Yes<br>No<br>Unclear	Yes<br>No<br>Unclear	**Low**<br>**High**
O'Carroll et al, 2011	Yes<br>No<br>Unclear	Yes<br>No<br>Unclear	Yes<br>No<br>NA	Yes<br>No<br>Unclear	Yes<br>No<br>Unclear	Yes<br>No<br>Unclear	Yes<br>No<br>Unclear	**Low**<br>**High**
Østergaard et al, 2012	Yes<br>No<br>Unclear	Yes<br>No<br>Unclear	Yes<br>No<br>NA	Yes<br>No<br>Unclear	Yes<br>No<br>Unclear	Yes<br>No<br>Unclear	Yes<br>No<br>Unclear	**Low**<br>**High**
Østergaard et al, 2014	Yes<br>No<br>Unclear	Yes<br>No<br>Unclear	Yes<br>No<br>NA	Yes<br>No<br>Unclear	Yes<br>No<br>Unclear	Yes<br>No<br>Unclear	Yes<br>No<br>Unclear	**Low**<br>**High**
Rodriguez et al, 2011	Yes<br>No<br>Unclear	Yes<br>No<br>Unclear	Yes<br>No<br>NA	Yes<br>No<br>Unclear	Yes<br>No<br>Unclear	Yes<br>No<br>Unclear	Yes<br>No<br>Unclear	**Low**<br>**High**
Sappok et al, 2001	Yes<br>No<br>Unclear	Yes<br>No<br>Unclear	Yes<br>No<br>NA	Yes<br>No<br>Unclear	Yes<br>No<br>Unclear	Yes<br>No<br>Unclear	Yes<br>No<br>Unclear	**Low**<br>**High**
Sjölander et al, 2012	Yes<br>No<br>Unclear	Yes<br>No<br>Unclear	Yes<br>No<br>NA	Yes<br>No<br>Unclear	Yes<br>No<br>Unclear	Yes<br>No<br>Unclear	Yes<br>No<br>Unclear	**Low**<br>**High**
Sjölander et al, 2013	Yes	Yes	Yes	Yes	Yes	Yes	Yes	**Low**

	No<br>Unclear	No<br>Unclear	No<br>NA	No<br>Unclear	No<br>Unclear	No<br>Unclear	No<br>Unclear	High
Thrift et al, 2014	Yes<br>No<br>Unclear	Yes<br>No<br>Unclear	Yes<br>No<br>NA	Yes<br>No<br>Unclear	Yes<br>No<br>Unclear	Yes<br>No<br>Unclear	Yes<br>No<br>Unclear	**Low**<br>High
Wang et al, 2006	Yes<br>No<br>Unclear	Yes<br>No<br>Unclear	Yes<br>No<br>NA	Yes<br>No<br>Unclear	Yes<br>No<br>Unclear	Yes<br>No<br>Unclear	Yes<br>No<br>Unclear	**Low**<br>High
Weimar et al, 2008	Yes<br>No<br>Unclear	Yes<br>No<br>Unclear	Yes<br>No<br>NA	Yes<br>No<br>Unclear	Yes<br>No<br>Unclear	Yes<br>No<br>Unclear	Yes<br>No<br>Unclear	**Low**<br>High
Xu et al, 2013	Yes<br>No<br>Unclear	Yes<br>No<br>Unclear	Yes<br>No<br>NA	Yes<br>No<br>Unclear	Yes<br>No<br>Unclear	Yes<br>No<br>Unclear	Yes<br>No<br>Unclear	**Low**<br>High

NA = Not Applicable.
